# Homeownership Among Older Adults: Source of Stability or Stress?

**DOI:** 10.1093/geroni/igaa057.2417

**Published:** 2020-12-16

**Authors:** Jennifer Molinsky, Christopher Herbert

**Affiliations:** 1 Harvard University, Cambridge, Massachusetts, United States; 2 Harvard university, Cambridge, Massachusetts, United States

## Abstract

For older adults, homeownership can be an important source of housing stability and personal wealth that can be tapped in later life, including for long-term care. Nonetheless, owning a home is not without physical and financial challenges for aging households, and many owners are reluctant to take advantage of housing equity later in life. This paper reviews the conditions of older homeowners to assess the degree to which owning a home is—or is not—associated with financial security and housing stability. We review trends in homeowning among older adults, including differences by race/ethnicity and income. We then describe the extent of housing affordability challenges among homeowners, the degree of mortgage indebtedness, and the extent and use of housing equity. Finally, we examine issues related to housing quality and accessibility. The paper concludes with a discussion of policy implications.

